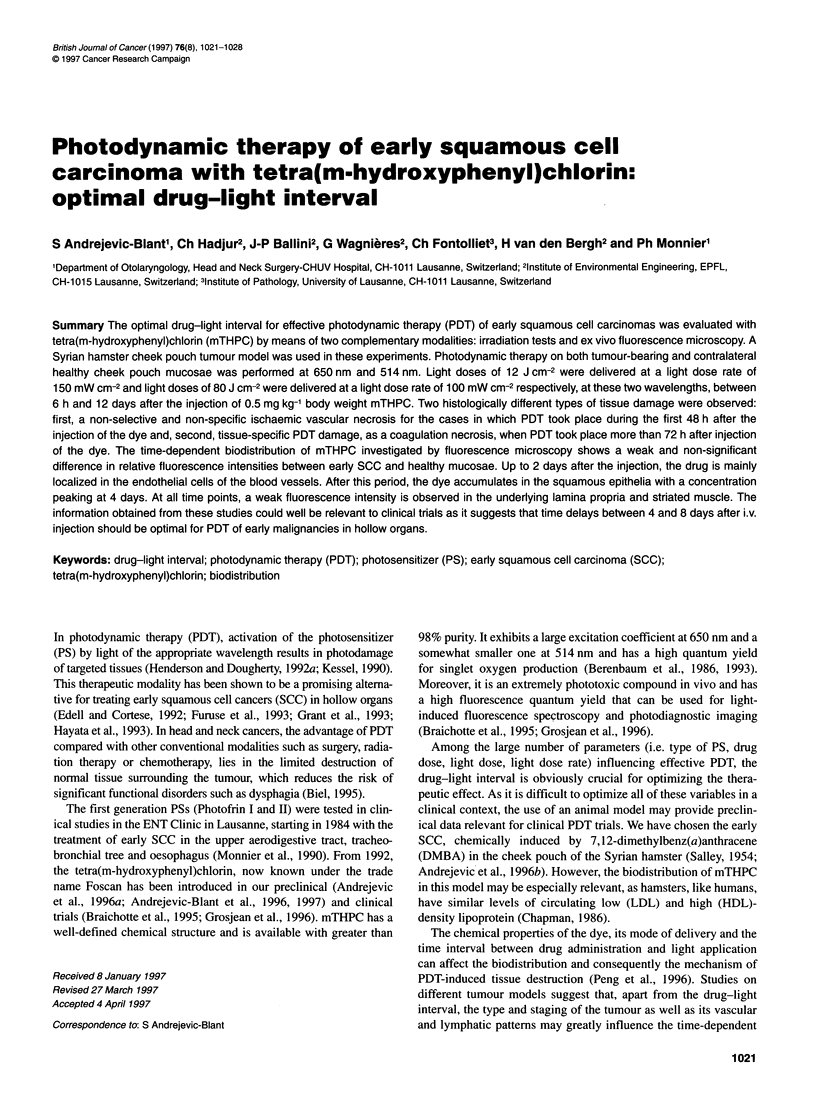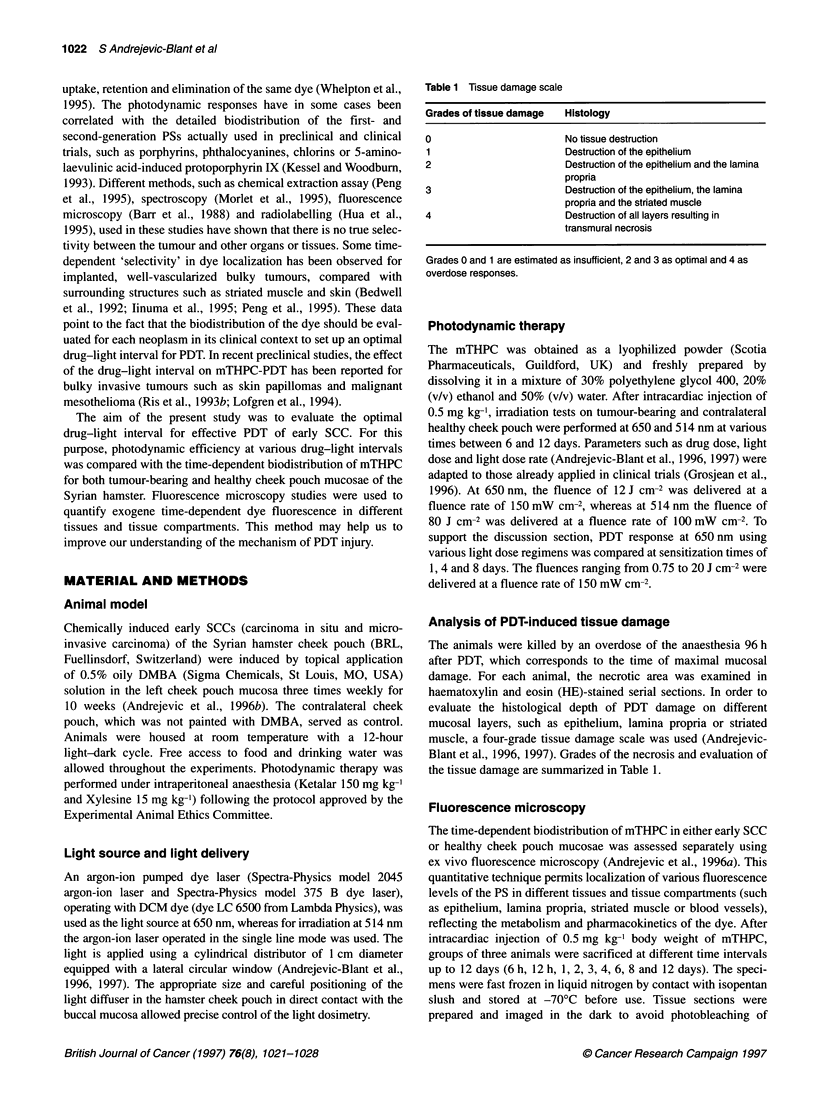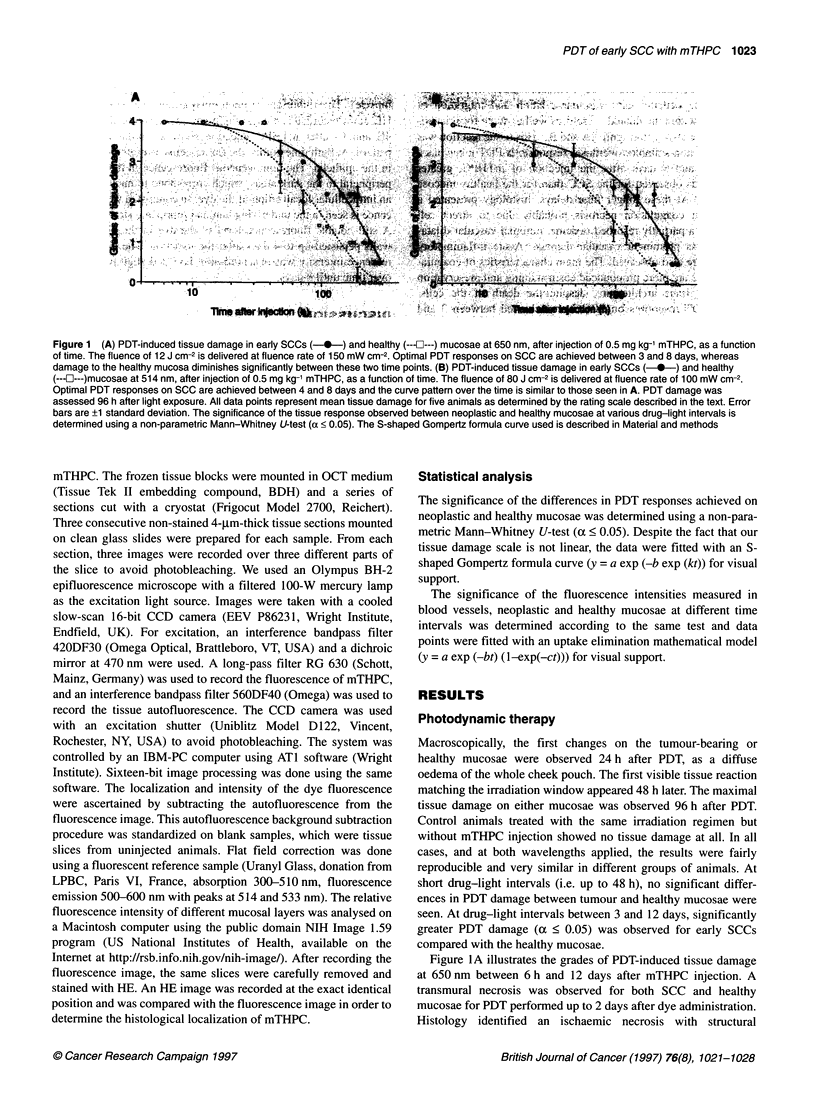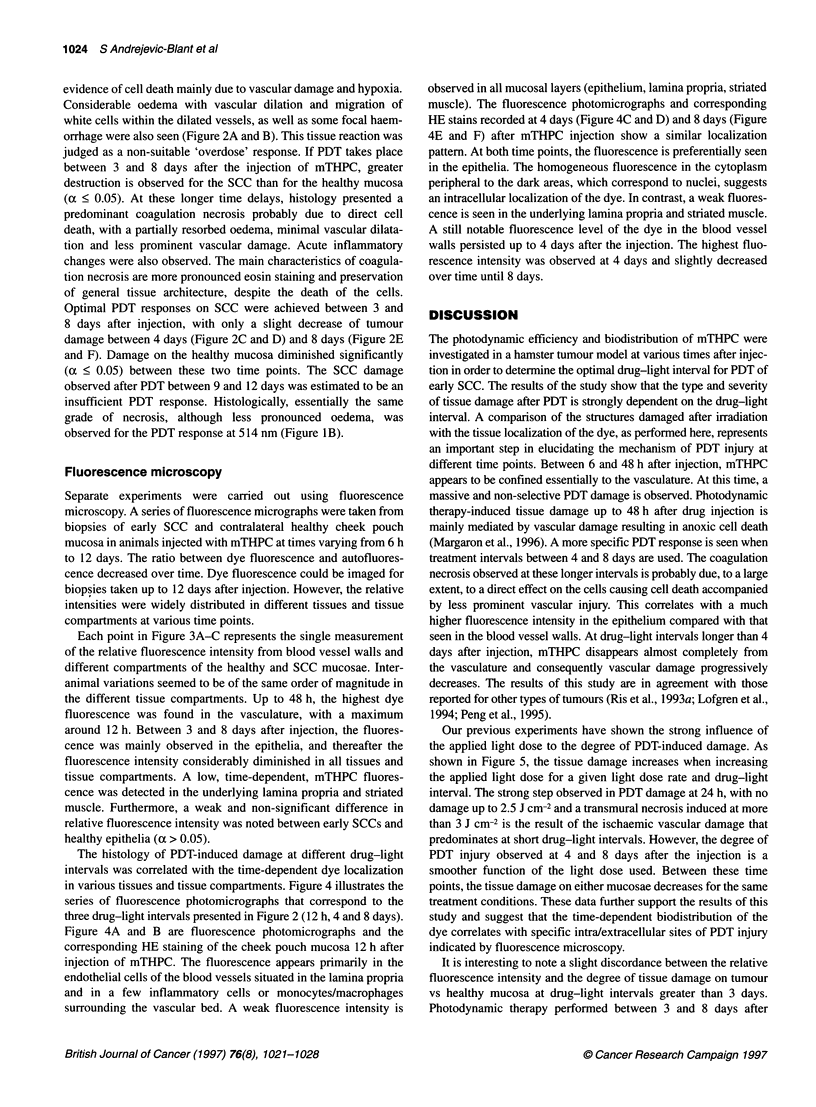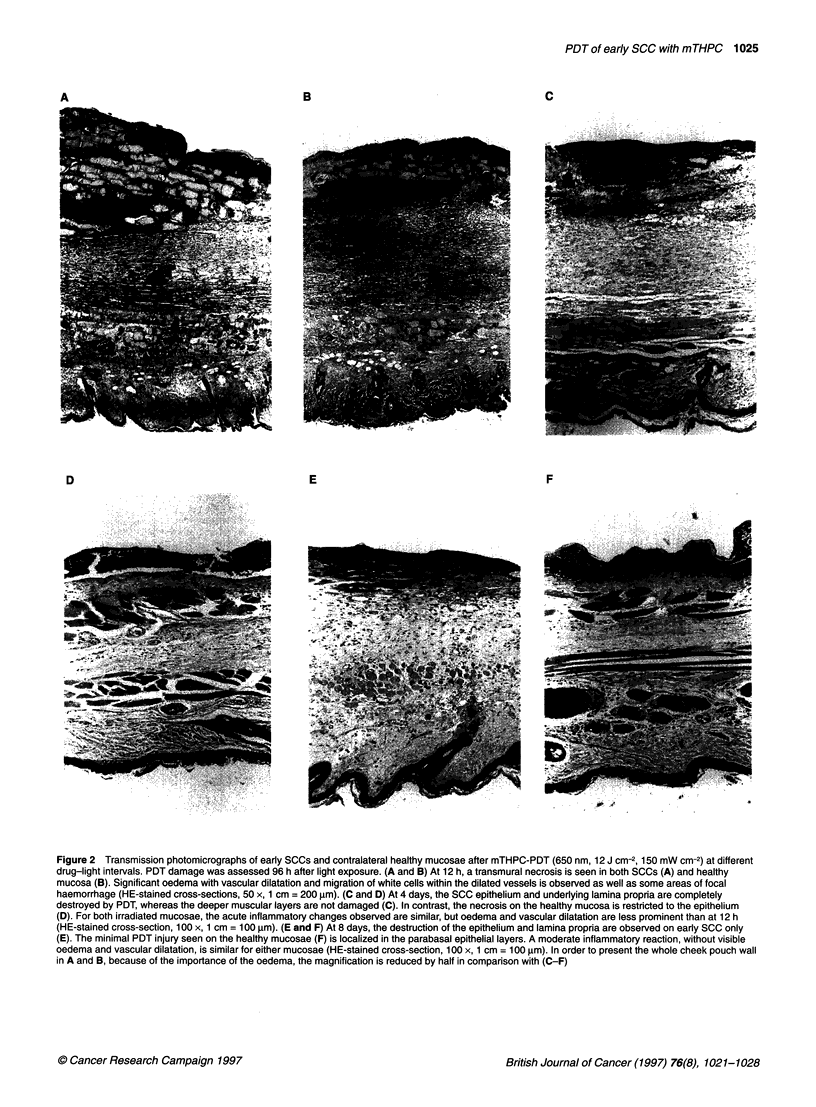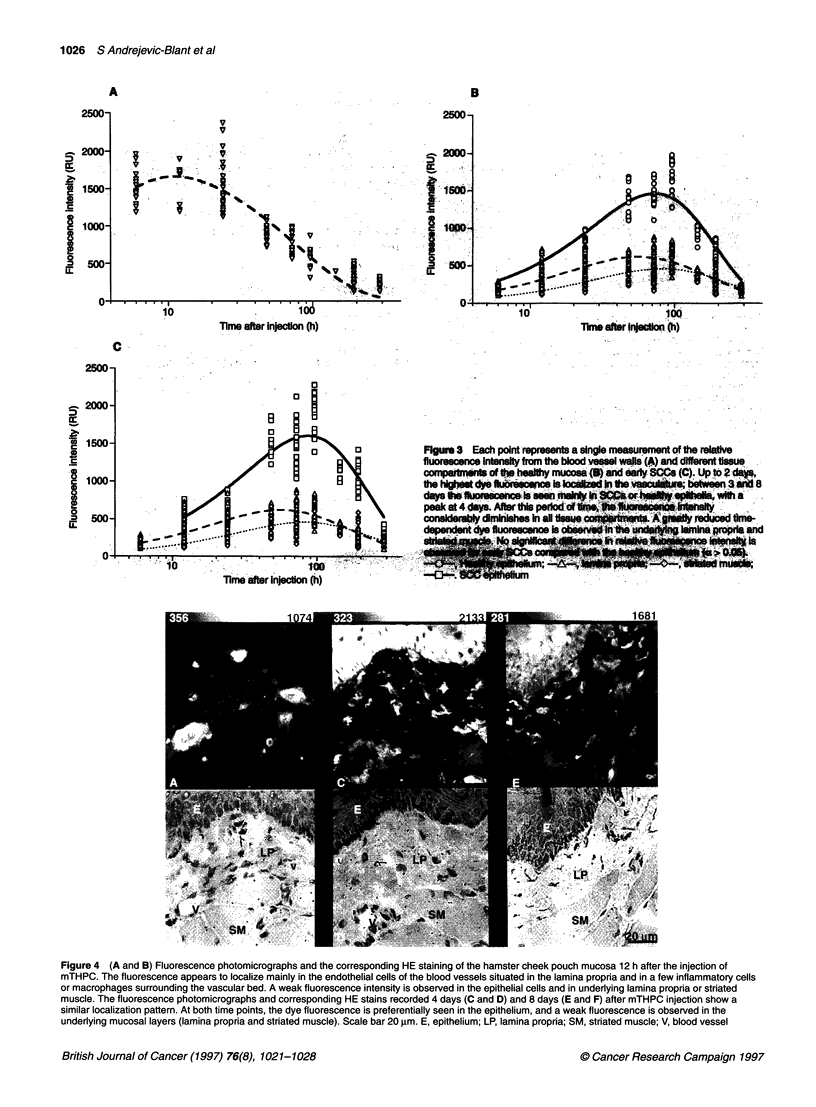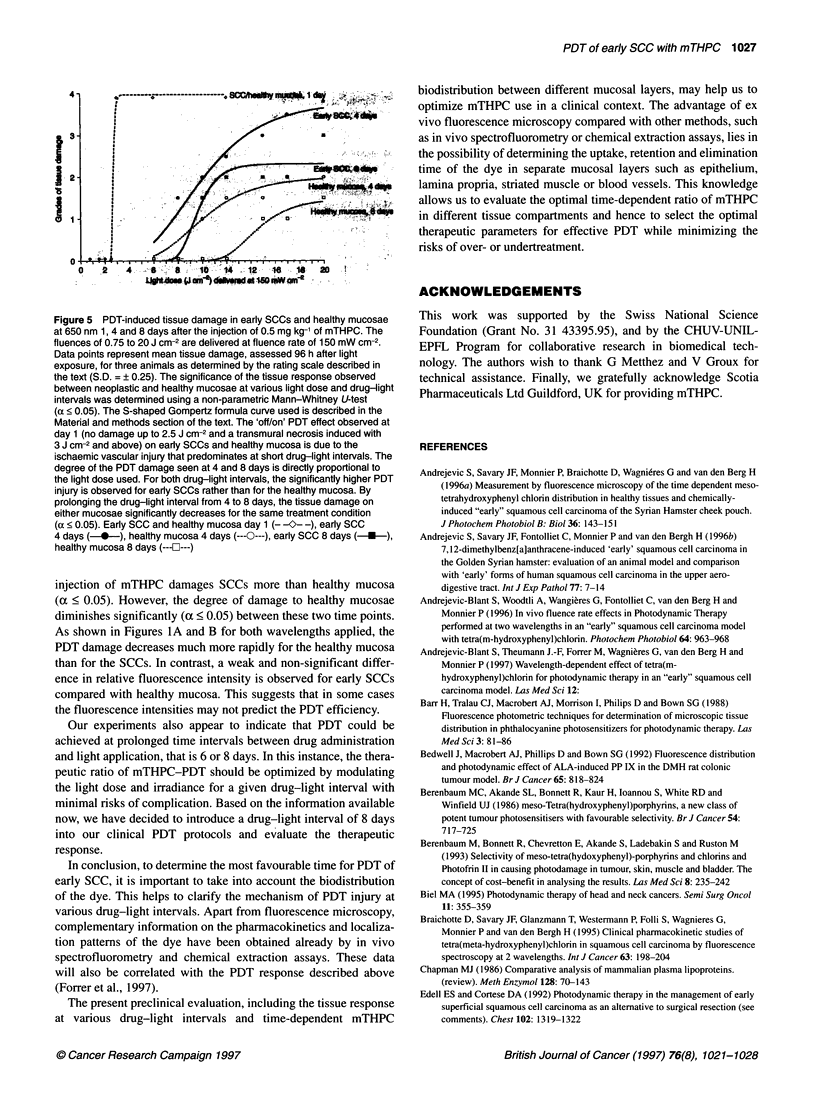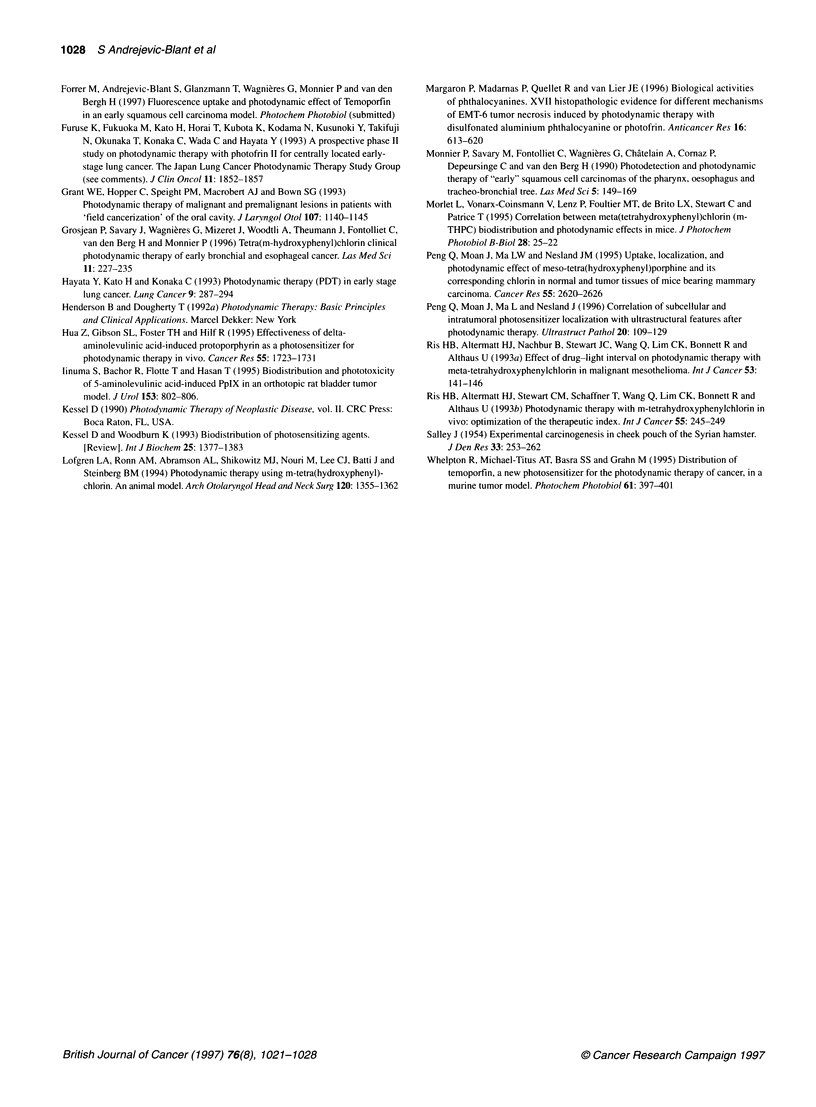# Photodynamic therapy of early squamous cell carcinoma with tetra(m-hydroxyphenyl)chlorin: optimal drug-light interval.

**DOI:** 10.1038/bjc.1997.502

**Published:** 1997

**Authors:** S. Andrejevic-Blant, C. Hadjur, J. P. Ballini, G. WagniÃ¨res, C. Fontolliet, H. van den Bergh, P. Monnier

**Affiliations:** Department of Otolaryngology, Head and Neck Surgery-CHUV Hospital, Lausanne, Switzerland.

## Abstract

**Images:**


British Journal of Cancer (1997) 76(8), 1021–1028
© 1997 Cancer Research Campaign

### Photodynamic therapy of early squamous cell carcinoma with tetra(m-hydroxyphenyl)chlorin: optimal drug–light interval

S Andrejevic-Blant[1], Ch Hadjur[2], J-P Ballini[2], G Wagnières[2], Ch Fontolliet[3], H van den Bergh[2] and Ph Monnier[1]

[1]Department of Otolaryngology, Head and Neck Surgery-CHUV Hospital, CH-1011 Lausanne, Switzerland; [2]Institute of Environmental Engineering, EPFL, CH-1015 Lausanne, Switzerland; [3]Institute of Pathology, University of Lausanne, CH-1011 Lausanne, Switzerland

**Summary** The optimal drug–light interval for effective photodynamic therapy (PDT) of early squamous cell carcinomas was evaluated with tetra(m-hydroxyphenyl)chlorin (mTHPC) by means of two complementary modalities: irradiation tests and ex vivo fluorescence microscopy. A Syrian hamster cheek pouch tumour model was used in these experiments. Photodynamic therapy on both tumour-bearing and contralateral healthy cheek pouch mucosae was performed at 650 nm and 514 nm. Light doses of 12 J $cm^{-2}$ were delivered at a light dose rate of 150 mW $cm^{-2}$ and light doses of 80 J $cm^{-2}$ were delivered at a light dose rate of 100 mW $cm^{-2}$ respectively, at these two wavelengths, between 6 h and 12 days after the injection of 0.5 mg $kg^{-1}$ body weight mTHPC. Two histologically different types of tissue damage were observed: first, a non-selective and non-specific ischaemic vascular necrosis for the cases in which PDT took place during the first 48 h after the injection of the dye and, second, tissue-specific PDT damage, as a coagulation necrosis, when PDT took place more than 72 h after injection of the dye. The time-dependent biodistribution of mTHPC investigated by fluorescence microscopy shows a weak and non-significant difference in relative fluorescence intensities between early SCC and healthy mucosae. Up to 2 days after the injection, the drug is mainly localized in the endothelial cells of the blood vessels. After this period, the dye accumulates in the squamous epithelia with a concentration peaking at 4 days. At all time points, a weak fluorescence intensity is observed in the underlying lamina propria and striated muscle. The information obtained from these studies could well be relevant to clinical trials as it suggests that time delays between 4 and 8 days after i.v. injection should be optimal for PDT of early malignancies in hollow organs.

**Keywords:** drug–light interval; photodynamic therapy (PDT); photosensitizer (PS); early squamous cell carcinoma (SCC); tetra(m-hydroxyphenyl)chlorin; biodistribution

In photodynamic therapy (PDT), activation of the photosensitizer (PS) by light of the appropriate wavelength results in photodamage of targeted tissues (Henderson and Dougherty, 1992*a*; Kessel, 1990). This therapeutic modality has been shown to be a promising alternative for treating early squamous cell cancers (SCC) in hollow organs (Edell and Cortese, 1992; Furuse et al., 1993; Grant et al., 1993; Hayata et al., 1993). In head and neck cancers, the advantage of PDT compared with other conventional modalities such as surgery, radiation therapy or chemotherapy, lies in the limited destruction of normal tissue surrounding the tumour, which reduces the risk of significant functional disorders such as dysphagia (Biel, 1995).

The first generation PSs (Photofrin I and II) were tested in clinical studies in the ENT Clinic in Lausanne, starting in 1984 with the treatment of early SCC in the upper aerodigestive tract, tracheobronchial tree and oesophagus (Monnier et al., 1990). From 1992, the tetra(m-hydroxyphenyl)chlorin, now known under the trade name Foscan has been introduced in our preclinical (Andrejevic et al., 1996*a*; Andrejevic-Blant et al., 1996, 1997) and clinical trials (Braichotte et al., 1995; Grosjean et al., 1996). mTHPC has a well-defined chemical structure and is available with greater than 98% purity. It exhibits a large excitation coefficient at 650 nm and a somewhat smaller one at 514 nm and has a high quantum yield for singlet oxygen production (Berenbaum et al., 1986, 1993). Moreover, it is an extremely phototoxic compound in vivo and has a high fluorescence quantum yield that can be used for light-induced fluorescence spectroscopy and photodiagnostic imaging (Braichotte et al., 1995; Grosjean et al., 1996).

Among the large number of parameters (i.e. type of PS, drug dose, light dose, light dose rate) influencing effective PDT, the drug–light interval is obviously crucial for optimizing the therapeutic effect. As it is difficult to optimize all of these variables in a clinical context, the use of an animal model may provide preclinical data relevant for clinical PDT trials. We have chosen the early SCC, chemically induced by 7,12-dimethylbenz(*a*)anthracene (DMBA) in the cheek pouch of the Syrian hamster (Salley, 1954; Andrejevic et al., 1996*b*). However, the biodistribution of mTHPC in this model may be especially relevant, as hamsters, like humans, have similar levels of circulating low (LDL) and high (HDL)-density lipoprotein (Chapman, 1986).

The chemical properties of the dye, its mode of delivery and the time interval between drug administration and light application can affect the biodistribution and consequently the mechanism of PDT-induced tissue destruction (Peng et al., 1996). Studies on different tumour models suggest that, apart from the drug–light interval, the type and staging of the tumour as well as its vascular and lymphatic patterns may greatly influence the time-dependent

*Received 8 January 1997*
*Revised 27 March 1997*
*Accepted 4 April 1997*

*Correspondence to*: S Andrejevic-Blant uptake, retention and elimination of the same dye (Whelpton et al., 1995). The photodynamic responses have in some cases been correlated with the detailed biodistribution of the first- and second-generation PSs actually used in preclinical and clinical trials, such as porphyrins, phthalocyanines, chlorins or 5-aminolaevulinic acid-induced protoporphyrin IX (Kessel and Woodburn, 1993). Different methods, such as chemical extraction assay (Peng et al., 1995), spectroscopy (Morlet et al., 1995), fluorescence microscopy (Barr et al., 1988) and radiolabelling (Hua et al., 1995), used in these studies have shown that there is no true selectivity between the tumour and other organs or tissues. Some time-dependent 'selectivity' in dye localization has been observed for implanted, well-vascularized bulky tumours, compared with surrounding structures such as striated muscle and skin (Bedwell et al., 1992; Iinuma et al., 1995; Peng et al., 1995). These data point to the fact that the biodistribution of the dye should be evaluated for each neoplasm in its clinical context to set up an optimal drug–light interval for PDT. In recent preclinical studies, the effect of the drug–light interval on mTHPC-PDT has been reported for bulky invasive tumours such as skin papillomas and malignant mesothelioma (Ris et al., 1993*b*; Lofgren et al., 1994).

The aim of the present study was to evaluate the optimal drug–light interval for effective PDT of early SCC. For this purpose, photodynamic efficiency at various drug–light intervals was compared with the time-dependent biodistribution of mTHPC for both tumour-bearing and healthy cheek pouch mucosae of the Syrian hamster. Fluorescence microscopy studies were used to quantify exogene time-dependent dye fluorescence in different tissues and tissue compartments. This method may help us to improve our understanding of the mechanism of PDT injury.

### MATERIAL AND METHODS

### Animal model

Chemically induced early SCCs (carcinoma in situ and micro-invasive carcinoma) of the Syrian hamster cheek pouch (BRL, Fuellinsdorf, Switzerland) were induced by topical application of 0.5% oily DMBA (Sigma Chemicals, St Louis, MO, USA) solution in the left cheek pouch mucosa three times weekly for 10 weeks (Andrejevic et al., 1996*b*). The contralateral cheek pouch, which was not painted with DMBA, served as control. Animals were housed at room temperature with a 12-hour light–dark cycle. Free access to food and drinking water was allowed throughout the experiments. Photodynamic therapy was performed under intraperitoneal anaesthesia (Ketalar 150 mg $kg^{-1}$ and Xylesine 15 mg $kg^{-1}$) following the protocol approved by the Experimental Animal Ethics Committee.

### Light source and light delivery

An argon-ion pumped dye laser (Spectra-Physics model 2045 argon-ion laser and Spectra-Physics model 375 B dye laser), operating with DCM dye (dye LC 6500 from Lambda Physics), was used as the light source at 650 nm, whereas for irradiation at 514 nm the argon-ion laser operated in the single line mode was used. The light is applied using a cylindrical distributor of 1 cm diameter equipped with a lateral circular window (Andrejevic-Blant et al., 1996, 1997). The appropriate size and careful positioning of the light diffuser in the hamster cheek pouch in direct contact with the buccal mucosa allowed precise control of the light dosimetry.

**Table 1** Tissue damage scale

| Grades of tissue damage | Histology |
|---|---|
| 0 | No tissue destruction |
| 1 | Destruction of the epithelium |
| 2 | Destruction of the epithelium and the lamina propria |
| 3 | Destruction of the epithelium, the lamina propria and the striated muscle |
| 4 | Destruction of all layers resulting in transmural necrosis |

Grades 0 and 1 are estimated as insufficient, 2 and 3 as optimal and 4 as overdose responses.

### Photodynamic therapy

The mTHPC was obtained as a lyophilized powder (Scotia Pharmaceuticals, Guildford, UK) and freshly prepared by dissolving it in a mixture of 30% polyethylene glycol 400, 20% (v/v) ethanol and 50% (v/v) water. After intracardiac injection of 0.5 mg $kg^{-1}$, irradiation tests on tumour-bearing and contralateral healthy cheek pouch were performed at 650 and 514 nm at various times between 6 and 12 days. Parameters such as drug dose, light dose and light dose rate (Andrejevic-Blant et al., 1996, 1997) were adapted to those already applied in clinical trials (Grosjean et al., 1996). At 650 nm, the fluence of 12 J $cm^{-2}$ was delivered at a fluence rate of 150 mW $cm^{-2}$, whereas at 514 nm the fluence of 80 J $cm^{-2}$ was delivered at a fluence rate of 100 mW $cm^{-2}$. To support the discussion section, PDT response at 650 nm using various light dose regimens was compared at sensitization times of 1, 4 and 8 days. The fluences ranging from 0.75 to 20 J $cm^{-2}$ were delivered at a fluence rate of 150 mW $cm^{-2}$.

### Analysis of PDT-induced tissue damage

The animals were killed by an overdose of the anaesthesia 96 h after PDT, which corresponds to the time of maximal mucosal damage. For each animal, the necrotic area was examined in haematoxylin and eosin (HE)-stained serial sections. In order to evaluate the histological depth of PDT damage on different mucosal layers, such as epithelium, lamina propria or striated muscle, a four-grade tissue damage scale was used (Andrejevic-Blant et al., 1996, 1997). Grades of the necrosis and evaluation of the tissue damage are summarized in Table 1.

### Fluorescence microscopy

The time-dependent biodistribution of mTHPC in either early SCC or healthy cheek pouch mucosae was assessed separately using ex vivo fluorescence microscopy (Andrejevic et al., 1996*a*). This quantitative technique permits localization of various fluorescence levels of the PS in different tissues and tissue compartments (such as epithelium, lamina propria, striated muscle or blood vessels), reflecting the metabolism and pharmacokinetics of the dye. After intracardiac injection of 0.5 mg $kg^{-1}$ body weight of mTHPC, groups of three animals were sacrificed at different time intervals up to 12 days (6 h, 12 h, 1, 2, 3, 4, 6, 8 and 12 days). The specimens were fast frozen in liquid nitrogen by contact with isopentan slush and stored at –70°C before use. Tissue sections were prepared and imaged in the dark to avoid photobleaching of

 © Cancer Research Campaign 1997

**Figure 1** (**A**) PDT-induced tissue damage in early SCCs (—●—) and healthy (---□---) mucosae at 650 nm, after injection of 0.5 mg $kg^{-1}$ mTHPC, as a function of time. The fluence of 12 J $cm^{-2}$ is delivered at fluence rate of 150 mW $cm^{-2}$. Optimal PDT responses on SCC are achieved between 3 and 8 days, whereas damage to the healthy mucosa diminishes significantly between these two time points. (**B**) PDT-induced tissue damage in early SCCs (—●—) and healthy (---□---)mucosae at 514 nm, after injection of 0.5 mg $kg^{-1}$ mTHPC, as a function of time. The fluence of 80 J $cm^{-2}$ is delivered at fluence rate of 100 mW $cm^{-2}$. Optimal PDT responses on SCC are achieved between 4 and 8 days and the curve pattern over the time is similar to those seen in **A**. PDT damage was assessed 96 h after light exposure. All data points represent mean tissue damage for five animals as determined by the rating scale described in the text. Error bars are ±1 standard deviation. The significance of the tissue response observed between neoplastic and healthy mucosae at various drug–light intervals is determined using a non-parametric Mann–Whitney *U*-test ($\alpha \leq 0.05$). The S-shaped Gompertz formula curve used is described in Material and methods mTHPC. The frozen tissue blocks were mounted in OCT medium (Tissue Tek II embedding compound, BDH) and a series of sections cut with a cryostat (Frigocut Model 2700, Reichert). Three consecutive non-stained 4-μm-thick tissue sections mounted on clean glass slides were prepared for each sample. From each section, three images were recorded over three different parts of the slice to avoid photobleaching. We used an Olympus BH-2 epifluorescence microscope with a filtered 100-W mercury lamp as the excitation light source. Images were taken with a cooled slow-scan 16-bit CCD camera (EEV P86231, Wright Institute, Endfield, UK). For excitation, an interference bandpass filter 420DF30 (Omega Optical, Brattleboro, VT, USA) and a dichroic mirror at 470 nm were used. A long-pass filter RG 630 (Schott, Mainz, Germany) was used to record the fluorescence of mTHPC, and an interference bandpass filter 560DF40 (Omega) was used to record the tissue autofluorescence. The CCD camera was used with an excitation shutter (Uniblitz Model D122, Vincent, Rochester, NY, USA) to avoid photobleaching. The system was controlled by an IBM-PC computer using AT1 software (Wright Institute). Sixteen-bit image processing was done using the same software. The localization and intensity of the dye fluorescence were ascertained by subtracting the autofluorescence from the fluorescence image. This autofluorescence background subtraction procedure was standardized on blank samples, which were tissue slices from uninjected animals. Flat field correction was done using a fluorescent reference sample (Uranyl Glass, donation from LPBC, Paris VI, France, absorption 300–510 nm, fluorescence emission 500–600 nm with peaks at 514 and 533 nm). The relative fluorescence intensity of different mucosal layers was analysed on a Macintosh computer using the public domain NIH Image 1.59 program (US National Institutes of Health, available on the Internet at http://rsb.info.nih.gov/nih-image/). After recording the fluorescence image, the same slices were carefully removed and stained with HE. An HE image was recorded at the exact identical position and was compared with the fluorescence image in order to determine the histological localization of mTHPC.

### Statistical analysis

The significance of the differences in PDT responses achieved on neoplastic and healthy mucosae was determined using a non-parametric Mann–Whitney *U*-test ($\alpha \leq 0.05$). Despite the fact that our tissue damage scale is not linear, the data were fitted with an S-shaped Gompertz formula curve ($y = a \exp(-b \exp(kt))$) for visual support.

The significance of the fluorescence intensities measured in blood vessels, neoplastic and healthy mucosae at different time intervals was determined according to the same test and data points were fitted with an uptake elimination mathematical model ($y = a \exp(-bt)(1-\exp(-ct))$) for visual support.

### RESULTS

### Photodynamic therapy

Macroscopically, the first changes on the tumour-bearing or healthy mucosae were observed 24 h after PDT, as a diffuse oedema of the whole cheek pouch. The first visible tissue reaction matching the irradiation window appeared 48 h later. The maximal tissue damage on either mucosae was observed 96 h after PDT. Control animals treated with the same irradiation regimen but without mTHPC injection showed no tissue damage at all. In all cases, and at both wavelengths applied, the results were fairly reproducible and very similar in different groups of animals. At short drug–light intervals (i.e. up to 48 h), no significant differences in PDT damage between tumour and healthy mucosae were seen. At drug–light intervals between 3 and 12 days, significantly greater PDT damage ($\alpha \leq 0.05$) was observed for early SCCs compared with the healthy mucosae.

Figure 1A illustrates the grades of PDT-induced tissue damage at 650 nm between 6 h and 12 days after mTHPC injection. A transmural necrosis was observed for both SCC and healthy mucosae for PDT performed up to 2 days after dye administration. Histology identified an ischaemic necrosis with structural

© Cancer Research Campaign 1997

evidence of cell death mainly due to vascular damage and hypoxia. Considerable oedema with vascular dilation and migration of white cells within the dilated vessels, as well as some focal haemorrhage were also seen (Figure 2A and B). This tissue reaction was judged as a non-suitable 'overdose' response. If PDT takes place between 3 and 8 days after the injection of mTHPC, greater destruction is observed for the SCC than for the healthy mucosa ($\alpha \leq 0.05$). At these longer time delays, histology presented a predominant coagulation necrosis probably due to direct cell death, with a partially resorbed oedema, minimal vascular dilatation and less prominent vascular damage. Acute inflammatory changes were also observed. The main characteristics of coagulation necrosis are more pronounced eosin staining and preservation of general tissue architecture, despite the death of the cells. Optimal PDT responses on SCC were achieved between 3 and 8 days after injection, with only a slight decrease of tumour damage between 4 days (Figure 2C and D) and 8 days (Figure 2E and F). Damage on the healthy mucosa diminished significantly ($\alpha \leq 0.05$) between these two time points. The SCC damage observed after PDT between 9 and 12 days was estimated to be an insufficient PDT response. Histologically, essentially the same grade of necrosis, although less pronounced oedema, was observed for the PDT response at 514 nm (Figure 1B).

### Fluorescence microscopy

Separate experiments were carried out using fluorescence microscopy. A series of fluorescence micrographs were taken from biopsies of early SCC and contralateral healthy cheek pouch mucosa in animals injected with mTHPC at times varying from 6 h to 12 days. The ratio between dye fluorescence and autofluorescence decreased over time. Dye fluorescence could be imaged for biopsies taken up to 12 days after injection. However, the relative intensities were widely distributed in different tissues and tissue compartments at various time points.

Each point in Figure 3A–C represents the single measurement of the relative fluorescence intensity from blood vessel walls and different compartments of the healthy and SCC mucosae. Interanimal variations seemed to be of the same order of magnitude in the different tissue compartments. Up to 48 h, the highest dye fluorescence was found in the vasculature, with a maximum around 12 h. Between 3 and 8 days after injection, the fluorescence was mainly observed in the epithelia, and thereafter the fluorescence intensity considerably diminished in all tissues and tissue compartments. A low, time-dependent, mTHPC fluorescence was detected in the underlying lamina propria and striated muscle. Furthermore, a weak and non-significant difference in relative fluorescence intensity was noted between early SCCs and healthy epithelia ($\alpha > 0.05$).

The histology of PDT-induced damage at different drug–light intervals was correlated with the time-dependent dye localization in various tissues and tissue compartments. Figure 4 illustrates the series of fluorescence photomicrographs that correspond to the three drug–light intervals presented in Figure 2 (12 h, 4 and 8 days). Figure 4A and B are fluorescence photomicrographs and the corresponding HE staining of the cheek pouch mucosa 12 h after injection of mTHPC. The fluorescence appears primarily in the endothelial cells of the blood vessels situated in the lamina propria and in a few inflammatory cells or monocytes/macrophages surrounding the vascular bed. A weak fluorescence intensity is observed in all mucosal layers (epithelium, lamina propria, striated muscle). The fluorescence photomicrographs and corresponding HE stains recorded at 4 days (Figure 4C and D) and 8 days (Figure 4E and F) after mTHPC injection show a similar localization pattern. At both time points, the fluorescence is preferentially seen in the epithelia. The homogeneous fluorescence in the cytoplasm peripheral to the dark areas, which correspond to nuclei, suggests an intracellular localization of the dye. In contrast, a weak fluorescence is seen in the underlying lamina propria and striated muscle. A still notable fluorescence level of the dye in the blood vessel walls persisted up to 4 days after the injection. The highest fluorescence intensity was observed at 4 days and slightly decreased over time until 8 days.

### DISCUSSION

The photodynamic efficiency and biodistribution of mTHPC were investigated in a hamster tumour model at various times after injection in order to determine the optimal drug–light interval for PDT of early SCC. The results of the study show that the type and severity of tissue damage after PDT is strongly dependent on the drug–light interval. A comparison of the structures damaged after irradiation with the tissue localization of the dye, as performed here, represents an important step in elucidating the mechanism of PDT injury at different time points. Between 6 and 48 h after injection, mTHPC appears to be confined essentially to the vasculature. At this time, a massive and non-selective PDT damage is observed. Photodynamic therapy-induced tissue damage up to 48 h after drug injection is mainly mediated by vascular damage resulting in anoxic cell death (Margaron et al., 1996). A more specific PDT response is seen when treatment intervals between 4 and 8 days are used. The coagulation necrosis observed at these longer intervals is probably due, to a large extent, to a direct effect on the cells causing cell death accompanied by less prominent vascular injury. This correlates with a much higher fluorescence intensity in the epithelium compared with that seen in the blood vessel walls. At drug–light intervals longer than 4 days after injection, mTHPC disappears almost completely from the vasculature and consequently vascular damage progressively decreases. The results of this study are in agreement with those reported for other types of tumours (Ris et al., 1993*a*; Lofgren et al., 1994; Peng et al., 1995).

Our previous experiments have shown the strong influence of the applied light dose to the degree of PDT-induced damage. As shown in Figure 5, the tissue damage increases when increasing the applied light dose for a given light dose rate and drug–light interval. The strong step observed in PDT damage at 24 h, with no damage up to 2.5 J $cm^{-2}$ and a transmural necrosis induced at more than 3 J $cm^{-2}$ is the result of the ischaemic vascular damage that predominates at short drug–light intervals. However, the degree of PDT injury observed at 4 and 8 days after the injection is a smoother function of the light dose used. Between these time points, the tissue damage on either mucosae decreases for the same treatment conditions. These data further support the results of this study and suggest that the time-dependent biodistribution of the dye correlates with specific intra/extracellular sites of PDT injury indicated by fluorescence microscopy.

It is interesting to note a slight discordance between the relative fluorescence intensity and the degree of tissue damage on tumour vs healthy mucosa at drug–light intervals greater than 3 days. Photodynamic therapy performed between 3 and 8 days after

 © Cancer Research Campaign 1997

**Figure 2** Transmission photomicrographs of early SCCs and contralateral healthy mucosae after mTHPC-PDT (650 nm, 12 J $cm^{-2}$, 150 mW $cm^{-2}$) at different drug–light intervals. PDT damage was assessed 96 h after light exposure. (**A** and **B**) At 12 h, a transmural necrosis is seen in both SCCs (**A**) and healthy mucosa (**B**). Significant oedema with vascular dilatation and migration of white cells within the dilated vessels is observed as well as some areas of focal haemorrhage (HE-stained cross-sections, 50 ×, 1 cm = 200 μm). (**C** and **D**) At 4 days, the SCC epithelium and underlying lamina propria are completely destroyed by PDT, whereas the deeper muscular layers are not damaged (**C**). In contrast, the necrosis on the healthy mucosa is restricted to the epithelium (**D**). For both irradiated mucosae, the acute inflammatory changes observed are similar, but oedema and vascular dilatation are less prominent than at 12 h (HE-stained cross-section, 100 ×, 1 cm = 100 μm). (**E and F**) At 8 days, the destruction of the epithelium and lamina propria are observed on early SCC only (**E**). The minimal PDT injury seen on the healthy mucosae (**F**) is localized in the parabasal epithelial layers. A moderate inflammatory reaction, without visible oedema and vascular dilatation, is similar for either mucosae (HE-stained cross-section, 100 ×, 1 cm = 100 μm). In order to present the whole cheek pouch wall in **A** and **B**, because of the importance of the oedema, the magnification is reduced by half in comparison with (**C–F**)

© Cancer Research Campaign 1997

**Figure 4** (**A** and **B**) Fluorescence photomicrographs and the corresponding HE staining of the hamster cheek pouch mucosa 12 h after the injection of mTHPC. The fluorescence appears to localize mainly in the endothelial cells of the blood vessels situated in the lamina propria and in a few inflammatory cells or macrophages surrounding the vascular bed. A weak fluorescence intensity is observed in the epithelial cells and in underlying lamina propria or striated muscle. The fluorescence photomicrographs and corresponding HE stains recorded 4 days (**C** and **D**) and 8 days (**E** and **F**) after mTHPC injection show a similar localization pattern. At both time points, the dye fluorescence is preferentially seen in the epithelium, and a weak fluorescence is observed in the underlying mucosal layers (lamina propria and striated muscle). Scale bar 20 µm. E, epithelium; LP, lamina propria; SM, striated muscle; V, blood vessel

*© Cancer Research Campaign 1997*

**Figure 5** PDT-induced tissue damage in early SCCs and healthy mucosae at 650 nm 1, 4 and 8 days after the injection of 0.5 mg kg$^{-1}$ of mTHPC. The fluences of 0.75 to 20 J cm$^{-2}$ are delivered at fluence rate of 150 mW cm$^{-2}$. Data points represent mean tissue damage, assessed 96 h after light exposure, for three animals as determined by the rating scale described in the text (S.D. = ± 0.25). The significance of the tissue response observed between neoplastic and healthy mucosae at various light dose and drug–light intervals was determined using a non-parametric Mann–Whitney *U*-test ($\alpha \leq 0.05$). The S-shaped Gompertz formula curve used is described in the Material and methods section of the text. The 'off/on' PDT effect observed at day 1 (no damage up to 2.5 J cm$^{-2}$ and a transmural necrosis induced with 3 J cm$^{-2}$ and above) on early SCCs and healthy mucosa is due to the ischaemic vascular injury that predominates at short drug–light intervals. The degree of the PDT damage seen at 4 and 8 days is directly proportional to the light dose used. For both drug–light intervals, the significantly higher PDT injury is observed for early SCCs rather than for the healthy mucosa. By prolonging the drug–light interval from 4 to 8 days, the tissue damage on either mucosae significantly decreases for the same treatment condition ($\alpha \leq 0.05$). Early SCC and healthy mucosa day 1 (– –◇– –), early SCC 4 days (—●—), healthy mucosa 4 days (---○---), early SCC 8 days (—■—), healthy mucosa 8 days (---□---)

injection of mTHPC damages SCCs more than healthy mucosa ($\alpha \leq 0.05$). However, the degree of damage to healthy mucosae diminishes significantly ($\alpha \leq 0.05$) between these two time points. As shown in Figures 1A and B for both wavelengths applied, the PDT damage decreases much more rapidly for the healthy mucosa than for the SCCs. In contrast, a weak and non-significant difference in relative fluorescence intensity is observed for early SCCs compared with healthy mucosa. This suggests that in some cases the fluorescence intensities may not predict the PDT efficiency.

Our experiments also appear to indicate that PDT could be achieved at prolonged time intervals between drug administration and light application, that is 6 or 8 days. In this instance, the therapeutic ratio of mTHPC–PDT should be optimized by modulating the light dose and irradiance for a given drug–light interval with minimal risks of complication. Based on the information available now, we have decided to introduce a drug–light interval of 8 days into our clinical PDT protocols and evaluate the therapeutic response.

In conclusion, to determine the most favourable time for PDT of early SCC, it is important to take into account the biodistribution of the dye. This helps to clarify the mechanism of PDT injury at various drug–light intervals. Apart from fluorescence microscopy, complementary information on the pharmacokinetics and localization patterns of the dye have been obtained already by in vivo spectrofluorometry and chemical extraction assays. These data will also be correlated with the PDT response described above (Forrer et al., 1997).

The present preclinical evaluation, including the tissue response at various drug–light intervals and time-dependent mTHPC biodistribution between different mucosal layers, may help us to optimize mTHPC use in a clinical context. The advantage of ex vivo fluorescence microscopy compared with other methods, such as in vivo spectrofluorometry or chemical extraction assays, lies in the possibility of determining the uptake, retention and elimination time of the dye in separate mucosal layers such as epithelium, lamina propria, striated muscle or blood vessels. This knowledge allows us to evaluate the optimal time-dependent ratio of mTHPC in different tissue compartments and hence to select the optimal therapeutic parameters for effective PDT while minimizing the risks of over- or undertreatment.

### ACKNOWLEDGEMENTS

This work was supported by the Swiss National Science Foundation (Grant No. 31 43395.95), and by the CHUV-UNIL-EPFL Program for collaborative research in biomedical technology. The authors wish to thank G Metthez and V Groux for technical assistance. Finally, we gratefully acknowledge Scotia Pharmaceuticals Ltd Guildford, UK for providing mTHPC.

### REFERENCES

Andrejevic S, Savary JF, Monnier P, Braichotte D, Wagniéres G and van den Berg H (1996*a*) Measurement by fluorescence microscopy of the time dependent meso-tetrahydroxyphenyl chlorin distribution in healthy tissues and chemically-induced "early" squamous cell carcinoma of the Syrian Hamster cheek pouch. *J Photochem Photobiol B: Biol* **36**: 143–151

Andrejevic S, Savary JF, Fontolliet C, Monnier P and van den Bergh H (1996*b*) 7,12-dimethylbenz[a]anthracene-induced 'early' squamous cell carcinoma in the Golden Syrian hamster: evaluation of an animal model and comparison with 'early' forms of human squamous cell carcinoma in the upper aero-digestive tract. *Int J Exp Pathol* **77**: 7–14

Andrejevic-Blant S, Woodtli A, Wangières G, Fontolliet C, van den Berg H and Monnier P (1996) In vivo fluence rate effects in Photodynamic Therapy performed at two wavelengths in an "early" squamous cell carcinoma model with tetra(m-hydroxyphenyl)chlorin. *Photochem Photobiol* **64**: 963–968

Andrejevic-Blant S, Theumann J.-F, Forrer M, Wagnières G, van den Berg H and Monnier P (1997) Wavelength-dependent effect of tetra(m-hydroxyphenyl)chlorin for photodynamic therapy in an "early" squamous cell carcinoma model. *Las Med Sci* **12**:

Barr H, Tralau CJ, Macrobert AJ, Morrison I, Philips D and Bown SG (1988) Fluorescence photometric techniques for determination of microscopic tissue distribution in phthalocyanine photosensitizers for photodynamic therapy. *Las Med Sci* **3**: 81–86

Bedwell J, Macrobert AJ, Phillips D and Bown SG (1992) Fluorescence distribution and photodynamic effect of ALA-induced PP IX in the DMH rat colonic tumour model. *Br J Cancer* **65**: 818–824

Berenbaum MC, Akande SL, Bonnett R, Kaur H, Ioannou S, White RD and Winfield UJ (1986) meso-Tetra(hydroxyphenyl)porphyrins, a new class of potent tumour photosensitisers with favourable selectivity. *Br J Cancer* **54**: 717–725

Berenbaum M, Bonnett R, Chevretton E, Akande S, Ladebakin S and Ruston M (1993) Selectivity of meso-tetra(hydoxyphenyl)-porphyrins and chlorins and Photofrin II in causing photodamage in tumour, skin, muscle and bladder. The concept of cost–benefit in analysing the results. *Las Med Sci* **8**: 235–242

Biel MA (1995) Photodynamic therapy of head and neck cancers. *Semi Surg Oncol* **11**: 355–359

Braichotte D, Savary JF, Glanzmann T, Westermann P, Folli S, Wagnieres G, Monnier P and van den Bergh H (1995) Clinical pharmacokinetic studies of tetra(meta-hydroxyphenyl)chlorin in squamous cell carcinoma by fluorescence spectroscopy at 2 wavelengths. *Int J Cancer* **63**: 198–204

Chapman MJ (1986) Comparative analysis of mammalian plasma lipoproteins. (review). *Meth Enzymol* **128**: 70–143

Edell ES and Cortese DA (1992) Photodynamic therapy in the management of early superficial squamous cell carcinoma as an alternative to surgical resection (see comments). *Chest* **102**: 1319–1322

© Cancer Research Campaign 1997

Forrer M, Andrejevic-Blant S, Glanzmann T, Wagnières G, Monnier P and van den Bergh H (1997) Fluorescence uptake and photodynamic effect of Temoporfin in an early squamous cell carcinoma model. *Photochem Photobiol* (submitted)

Furuse K, Fukuoka M, Kato H, Horai T, Kubota K, Kodama N, Kusunoki Y, Takifuji N, Okunaka T, Konaka C, Wada C and Hayata Y (1993) A prospective phase II study on photodynamic therapy with photofrin II for centrally located early-stage lung cancer. The Japan Lung Cancer Photodynamic Therapy Study Group (see comments). *J Clin Oncol* **11**: 1852–1857

Grant WE, Hopper C, Speight PM, Macrobert AJ and Bown SG (1993) Photodynamic therapy of malignant and premalignant lesions in patients with 'field cancerization' of the oral cavity. *J Laryngol Otol* **107**: 1140–1145

Grosjean P, Savary J, Wagnières G, Mizeret J, Woodtli A, Theumann J, Fontolliet C, van den Berg H and Monnier P (1996) Tetra(m-hydroxyphenyl)chlorin clinical photodynamic therapy of early bronchial and esophageal cancer. *Las Med Sci* **11**: 227–235

Hayata Y, Kato H and Konaka C (1993) Photodynamic therapy (PDT) in early stage lung cancer. *Lung Cancer* **9**: 287–294

Henderson B and Dougherty T (1992*a*) *Photodynamic Therapy: Basic Principles and Clinical Applications*. Marcel Dekker: New York

Hua Z, Gibson SL, Foster TH and Hilf R (1995) Effectiveness of delta-aminolevulinic acid-induced protoporphyrin as a photosensitizer for photodynamic therapy in vivo. *Cancer Res* **55**: 1723–1731

Iinuma S, Bachor R, Flotte T and Hasan T (1995) Biodistribution and phototoxicity of 5-aminolevulinic acid-induced PpIX in an orthotopic rat bladder tumor model. *J Urol* **153**: 802–806.

Kessel D (1990) *Photodynamic Therapy of Neoplastic Disease*, vol. II. CRC Press: Boca Raton, FL, USA.

Kessel D and Woodburn K (1993) Biodistribution of photosensitizing agents. [Review]. *Int J Biochem* **25**: 1377–1383

Lofgren LA, Ronn AM, Abramson AL, Shikowitz MJ, Nouri M, Lee CJ, Batti J and Steinberg BM (1994) Photodynamic therapy using m-tetra(hydroxyphenyl)-chlorin. An animal model. *Arch Otolaryngol Head and Neck Surg* **120**: 1355–1362

Margaron P, Madarnas P, Quellet R and van Lier JE (1996) Biological activities of phthalocyanines. XVII histopathologic evidence for different mechanisms of EMT-6 tumor necrosis induced by photodynamic therapy with disulfonated aluminium phthalocyanine or photofrin. *Anticancer Res* **16**: 613–620

Monnier P, Savary M, Fontolliet C, Wagnières G, Châtelain A, Cornaz P, Depeursinge C and van den Berg H (1990) Photodetection and photodynamic therapy of "early" squamous cell carcinomas of the pharynx, oesophagus and tracheo-bronchial tree. *Las Med Sci* **5**: 149–169

Morlet L, Vonarx-Coinsmann V, Lenz P, Foultier MT, de Brito LX, Stewart C and Patrice T (1995) Correlation between meta(tetrahydroxyphenyl)chlorin (m-THPC) biodistribution and photodynamic effects in mice. *J Photochem Photobiol B-Biol* **28**: 25–22

Peng Q, Moan J, Ma LW and Nesland JM (1995) Uptake, localization, and photodynamic effect of meso-tetra(hydroxyphenyl)porphine and its corresponding chlorin in normal and tumor tissues of mice bearing mammary carcinoma. *Cancer Res* **55**: 2620–2626

Peng Q, Moan J, Ma L and Nesland J (1996) Correlation of subcellular and intratumoral photosensitizer localization with ultrastructural features after photodynamic therapy. *Ultrastruct Pathol* **20**: 109–129

Ris HB, Altermatt HJ, Nachbur B, Stewart JC, Wang Q, Lim CK, Bonnett R and Althaus U (1993*a*) Effect of drug–light interval on photodynamic therapy with meta-tetrahydroxyphenylchlorin in malignant mesothelioma. *Int J Cancer* **53**: 141–146

Ris HB, Altermatt HJ, Stewart CM, Schaffner T, Wang Q, Lim CK, Bonnett R and Althaus U (1993*b*) Photodynamic therapy with m-tetrahydroxyphenylchlorin in vivo: optimization of the therapeutic index. *Int J Cancer* **55**: 245–249

Salley J (1954) Experimental carcinogenesis in cheek pouch of the Syrian hamster. *J Den Res* **33**: 253–262

Whelpton R, Michael-Titus AT, Basra SS and Grahn M (1995) Distribution of temoporfin, a new photosensitizer for the photodynamic therapy of cancer, in a murine tumor model. *Photochem Photobiol* **61**: 397–401

© Cancer Research Campaign 1997